# Improving mental health by improving the mental health literacy? Study protocol for a randomised controlled evaluation of an e-mental health application as a preventive intervention for adolescents and young adults

**DOI:** 10.1016/j.invent.2024.100733

**Published:** 2024-03-07

**Authors:** Olivia Krokos, Isabel Brandhorst, Lennart Seizer, Caterina Gawrilow, Johanna Löchner

**Affiliations:** a Department of Child and Adolescent Psychiatry, Psychosomatics and Psychotherapy, University Hospital, Tuebingen, Germany; b Department of School Psychology, Universität Tübingen, Germany; c German Center for Mental Health (DZPG), Germany

**Keywords:** mHealth, Mental health literacy, Adolescence, Prevention

## Abstract

**Background:**

From the age of 14, many adolescents enter a vulnerable developmental phase, with a sharp increase in mental illness at 16. The COVID19 pandemic has further exacerbated this issue. Hence, universal and easily accessible prevention in the young is needed. *E*-mental health interventions are on the rise due to numerous benefits such as potential low-costs, low-threshold and high scalability. However, effectiveness and acceptance of mobile health (mHealth) preventive interventions remain unresearched.

**Method:**

In a two-armed, randomised controlled study design adolescents and young adults from 14 years old will be recruited. Following an initial baseline assessment, they will be randomised to a) the intervention group (IG, *n* = 75), which will receive a mHealth intervention (the application ‘Mental Health Guide’, co-developed by lived experience experts) or b) the waiting list control group (CG, *n* = 75). Both groups will be followed up after 3 and 6 months following post assessment. We hypothesize an increase in mental health literacy in the IG compared to the CG for post and follow-up assessment (primary outcome: Mental Health Literacy Scale). In addition, we expect an improvement in mental health and psychological well-being, improved emotion regulation, reduced psychological distress, as well as good quality ratings in usability and acceptance in the use of the ‘Mental Health Guide’ We performed multiple simulations of possible outcome scenarios, incorporating an array of factors, to generate realistic datasets and obtain accurate estimates of statistical power.

**Conclusion:**

As a first-of-its-kind in this field, this study investigates whether a mHealth intervention based on mental health literacy may improve the mental health literacy and further aspects of psychological functioning of young people in a vulnerable phase. Furthermore, the results promise to provide important knowledge of how universal prevention may be implemented with low costs for diverse populations.

**Trial registration:**

This trial was registered in the DRKS register (DRKS-ID: DRKS00031810) on 23 June 2023.

## Highlights

• Adolescents are at high risk of developing a mental illness, hence, prevention is needed.
• Improving Mental Health Literacy could help strengthen mental health in adolescents.
• This study will test whether the mHealth application “Mental Health Guide” is effective.
• The article describes the content of the application and the simulation of the results.

## 1 Introduction

Most mental disorders have an onset in childhood and adolescence with a global peak for the age of onset of any mental disorder at 14.5 years on average (Solmi et al., 2022). The COVID19 pandemic has contributed to further deterioration in adolescent mental health (Plötner et al., 2022). Accordingly, prevention of mental disorders at an early stage and to strengthen mental health particularly in the young is crucial. Already in 2004, the World Health Organization (WHO) issued an appeal and defined prevention as a ‘public health priority’ (World Health Organization, 2004). Hence, preventive measures in adolescence are needed to encounter the trend of rise in mental health problems at a young age.

According to the WHO, mental health is ‘a state of well-being in which the individual realizes his or her own abilities, can cope with the normal stresses of life, can work productively and fruitfully, and is able to make a contribution to his or her community’ (World Health Organization, 2004). Good well-being is essential, especially in young adulthood, for academic achievements, quality of life, and social interaction (Fuchs and Karwautz, 2017). Stress, anxiety, and emotion regulation problems, on the other hand, are the most common risk factors for the development of mental disorders in adolescents and young adults (Patel et al., 2007). One way of promoting mental health is educating people about such risk and protective factors that influence the development of mental disorders (Fuchs and Karwautz, 2017). Knowledge about possible risk and protective factors is considered part of mental health literacy (MHL). MHL is defined as knowledge and belief about mental health problems and is subdivided into the following characteristics: the ability to recognize specific disorders, knowledge of mental health information sources, knowledge of risk and protective factors, knowledge of self-help, and knowledge of available professional help and attitudes that promote recognition and help-seeking behaviors (Jorm, 2012). Lack of knowledge, prejudice, and stigmatization of mental disorders lead to avoidance of seeking psychotherapeutic services (Henderson et al., 2013). Good MHL enhances the search for and use of preventative services (Jorm et al., 1997).

An increasing number of recent studies on prevention with different content orientations indicate positive effects on the onset and symptoms of mental illness (Weisz et al., 2005; Brent and Melhem, 2023) and involve strengthening mental health and resilience (Hetrick et al., 2016). To strengthen the MHL a broad and global preventive approach is recommended due to its general educational aim. Preventions that target the whole population are universal interventions. Selective and indicative preventions are targeting subgroups with high-risk factors (selective) or first symptoms (indicative) of mental illness (Siepmann, 2012). Meta-analyses showed that universal prevention can be effective in preventing mental disorders (Ise et al., 2012; Hetrick et al., 2016) and increase knowledge about mental disorders (Schiller et al., 2014). In addition, universal prevention can also contribute to a more health-conscious lifestyle in general, as well as to the maintenance and improvement of occupational performance (Jorm, 2012). Thus, research indicates that promoting mental health already in adolescence through universal prevention is a useful approach. However, despite the advantages of high scalability, low cost and reaching out to everyone, universal prevention was found to have lower effect sizes and fewer long-lasting effects compared to selective and indicative prevention (Hetrick et al., 2016; Loechner et al., 2018), partly due to a wider distribution of answers in universal prevention. To further exacerbate the advantages of universal prevention, more research is needed to understand mechanisms of change and how populations can be reached to improve their mental health on an individual basis.

The achievements of digital technology may push universal prevention research to another level: based on the challenges during COVID19 pandemic and the fast-evolving digital changes in the last decade, mobile health (mHealth) based interventions are on the rise and hold promise to be effective also in the prevention of mental disorders. Adolescents and young adults are particularly media-savvy, with around 99 % of 12- to 19-year-olds owning a smartphone (Feierabend et al., 2023). The benefits of mHealth interventions are numerous: low-threshold access, geographically independent availability, potentially low operating costs (Paganini et al., 2018) temporal and locational flexibility, and promotion of self-determination (Robinson et al., 2016). Since the COVID19 pandemic, there has been even greater interest in and downloads of mHealth apps, with more than 69 % of 14- to 22-year-olds reporting the usage of mHealth apps (Rideout et al., 2021; Wang et al., 2023). In 2022, 54.546 mHealth Apps were offered in the Google Play Store and 41.517 mHealth Apps in the Apple App Store (Mikulic, 2022). Although the number of offered mental health apps and their use are increasing, comparatively few feasibility and efficacy studies address this subject (Larsen et al., 2019). Recent studies suggest that digital interventions are a generally appropriate approach and an additional resource in prevention efforts (Beames et al., 2023). Studies with quantitative data analyses have focused on the effectiveness of mHealth prevention for specific mental disorders such as depression, anxiety disorders (Torous et al., 2021), or substance abuse (Kazemi et al., 2017). To our knowledge, there is only one study on the effectiveness of mHealth universal prevention (Newbold et al., 2020). A challenge in the evaluation of apps as preventive measures is the low adherence together with high dropout rates (up to 47 %), especially in self-help apps (Linardon et al., 2019; Torous et al., 2020). In contrast, personal counseling or guided interventions are associated with lower dropout rates and higher compliance but not necessarily with higher effects (Moshe et al., 2021; Pauley et al., 2023). In order to increase adherence as much as possible and to keep dropout low, not only the quality of information, and acceptance of the intervention, but also the delivery method and the design of the app need to be investigated (Grist et al., 2017).

One mHealth intervention for implementing universal prevention of mental illness in young people is the ‘Mental Health Guide’ (MHG; for further information see also https://www.mentalhealthcrowd.de/was-wir-tun/mentalhealthguide/). The MHG is intended as a universal prevention tool to strengthen the mental health and improve the MHL. Although the MHG seems to be an attractive and suitable offering for the target group of adolescents, scientific studies that test both the usability and the effectiveness of the app are still pending. The present study aims to close this gap.

Primarily, we hypothesize that the use of the MHG leads to an increased MHL in adolescents and young adults aged 14 years and older. Secondly, we hypothesize that the use of the MHG will have a positive impact on mental health, including improved emotion regulation skills, reduced psychological distress, and improved psychological well-being. In addition, the usability and acceptance of the MHG as mHealth product will be investigated.

## 2 Materials and method

The study design is reported in line with the SPIRIT 2013 Statement (Standard Protocol Items: Recommendations for Interventional Trials; Chan et al., 2013), has received approval from the ethical committee of the Universitätsklinikum Tübingen (Ref no. 842/2022BO1, June 2023), and is in line with the Declaration of Helsinki (Anon, 2013), and has received approval from the German ministry of education (Ref. no. KM31-6499-3/115/3). The study was registered in the German register for clinical trials (DRKS-ID: DRKS00031810). The anonymized data and analysis codes will be uploaded in the open science framework (OSF: https://osf.io/4wh5x/?view_only=040375cd3a56472597e788b97e5b8f28).

### 2.1 Design

**Fig. 1:**
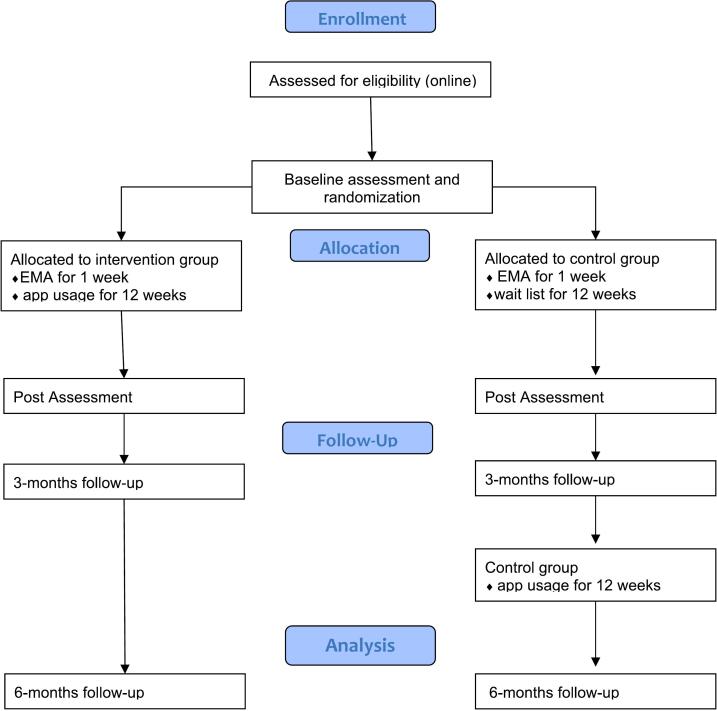
Overview of the MHG study design.

The study is a randomised controlled trial aiming to promote MHL and strengthen the mental health of *N* = 150 adolescents and young adults, as well as evaluating the usability and acceptance of the MHG. Fig. 1 provides an overview over the study design. In a parallel two-arm design, participants will be randomly assigned to the intervention group (IG, *n* = 75) or the waiting list control group (CG, n = 75) after a baseline online questionnaire by a computerised random generator, based on codes only. The IG will receive access to the MHG after a baseline measurement. The CG will receive access to the MHG after a post- and 3-month follow-up assessment. The relatively long waiting time of the CG until the intervention (6 months) is necessary as a comparison for the developments of the IG, but justifiable due to the preventive nature of the intervention. To capture and compare the ratings in usability and acceptance at the CG, further assessments will be administered at 6 months after the post-measurement. Additionally, to capture daily fluctuations in mood dynamics and emotion regulation, we carry out an ecological momentary assessment (EMA), to complement the pre-post-assessment with participants' experience in real-time (Schiffman et al., 2008).

### 2.2 Participants

Eligible participants are a) at least 14 years of age, b) own a smartphone (iOS or Android) with internet capability and c) have adequate German language skills according to their subjective assessment. Before participation, all participants must agree to the study and data protection conditions and provide written consent. For participants under the age of 18 years, the parental consent is also required.

As this is a primary prevention study, no further inclusion or exclusion criteria are applied. The results of the diagnostics are not evaluated at an individual level and the participants are not informed about their results or about the possible presence of mental abnormalities. The participants were informed of this as part of the study information. However, if participants recognize the need to seek professional help for a thorough diagnostic interview, participants are encouraged to do so.

Participants are recruited via high schools in the south of Germany (Baden-Wuerttemberg) and various social media channels. After completing the study, each participant will continue to receive free access to the MHG as well as an opportunity to take part in a voucher draw, unless they are students in a school in Baden-Wuerttemberg (participation requirement of ministry of education).

### 2.3 Procedure

School classes are informed about the study and the conditions of participation by the study staff. Interested students can provide consent directly via a QR code. Interested students under the age of 18 need to forward an information letter to their parents for the initial parental consent to the study. For participants over the age of 18 who are recruited via social media, all study information will be provided, and consent will be given online. The consent form of participants is automatically followed by the digital baseline survey. In addition, participants will be prompted for the EMA survey via smartphone twice a day (at 7 am and 9 pm) for one week as part of each assessment. After the baseline questionnaire (before the EMA survey), participants receive information whether they were randomised to either the IG or CG. As soon as the EMA survey is finished, the IG will receive access to the MHG. The IG may use the MHG at their discretion and will not be prompted to use it during the 12 weeks of use.

### 2.4 Interventions

#### 2.4.1 MHG

The MHG is a self-help e-learning course developed and distributed by a social company called the Mental Health Crowd. The app was developed amongst others by experts with lived experience of mental illness, psychologists, coaches and therapists. The motivation behind the co-development with lived experience experts and decisive for the selection of modules was to bring personal experiences into the designs in order to make the app more user-oriented. The study team was not and is not involved in the development or distribution of the app. The MHG can be used as a progressive web application on a smartphone, or as a desktop version via an Internet browser. In 12 modules, participants learn which factors influence mental health and how they can influence it. There is no predefined order in which modules should be worked through and by when to increase flexibility and address individual interests (see an overview of all modules displayed in Table 1 and in detail in supplemental 1). Users are informed that they should work independently on the topic that interests them most from an overview of the 12 modules. They are not given any specific recommendations. The content is presented to the user in a mixture of videos, texts, images, impulses, and quizzes. Personal counseling is not offered within the MHG due to limited resources. The processing period of the individual modules amounts to a suggested time of 12 weeks, so that each module can be sufficiently processed within one week. The processing period of a single module with its subsection varies between 20 and 60 min. However, when and for how long participants actually use the MHG is up to them personally and is not controlled or requested.

**Table 1:** Summary of the 12 MHG modules.

Module	Content
#1 – Body	Healthy nutrition, exercise, sleep, breathing, stimulants
#2 – Stress and resources	(Physical) effects of stress, handling stress
#3 – Here and now	Mindfulness, change in life
#4 – Circumstances	Finances and consumer goods, geographical and temporal circumstances, human, mindset
#5 – Emotional intelligence	Fundamental information of emotions, emotions in myself and others
#6 – Needs	Fundamental information of human needs, communication of needs
#7 – Relationships with others	Roles in everyday life, social skills, forgiving
#8 – Relationship with me	Self-care, beliefs
#9 – Crisis	Dealing with crises, exercise for coping strategies
#10 – Psychoeducation	Information on symptoms and diagnostics of mental disorders, therapies and other treatment options
#11 – Communication	Fundamental information of communication and active listening, how to communicate about mental health
#12 – Values, goals, purpose	What are values, how to achieve goals, linking between values, goals and purposes

#### 2.4.2 Control group

The control group does not receive an intervention between pre-measurement and 3-month follow-up. They will get access to the MHG between the 3-month and the 6-month follow-up.

### 2.5 Measures

Table 2 provides a list of all outcome measures.

**Table 2:** Summary of outcome measures.

Questionnaire	Baseline	EMA	Post	3 MFU	6 MFU
MHLS	X		X	X	X
SDQ	X		X	X	X
WHO-5	X		X	X	X
MDBF		X			
DERS-SF	X	X	X	X	X
RRS Brooding	X		X	X	X
PSS-4	X		X	X	X
MARS-G			X	X	X

Note. 3/6 MFU: 3/6 months follow-up*,* EMA: Ecological Momentary Assessment, MHLS: Mental Health Literacy Scale, MARS-G: Mobile App Rating Scale, SDQ: Strengths and Difficulties Questionnaire, DERS-SF: Difficulties in Emotion Regulation Scale -short form, RRS Brooding: Ruminative Response Scale Brooding, MDBF: Multidimensional Mood State Questionnaire, PSS-4: Perceived Stress Scale.

#### 2.5.1 Primary outcome

We expect the transfer of knowledge as part of the MHG to increase mental health literacy. Therefore, the primary outcomes are differences in the participants MHL and will be assessed with the Mental Health Literacy Scale (MHLS) (O’Connor and Casey, 2015). The MHLS is a self-report questionnaire in German language consisting of 36 items divided into five dimensions of MHL, which are assessed using different Likert scales. The scales “recognition of disorders” (8 items) and “knowledge of professional help available” (3 items) are rated on a 4-point Likert scale ranging from ‘1 = very unlikely’ to ‘4 = very likely’. The dimension “knowledge of risk factors and causes” (2 items) is rated on a 4-point-Likert scale ranging from ‘1 = very unhelpful’ to ‘4 = very helpful’. The dimension “knowledge of how to seek mental health information” (4 items) is rated on a 5-point-Likert scale ranging from ‘1 = strongly disagree’ to ‘5 = strongly agree’. The dimension “attitudes that promote recognition and appropriate help-seeking” (16 items) is rated on a 5-point-Likert scale ranging from ‘1 = definitely unwilling’ to ‘5 = definitely willing’. Overall, the MHLS showed good psychometric properties with an internal consistency of Cronbach's *α* = 0.873 (O’Connor and Casey, 2015).

#### 2.5.2 Secondary outcomes

Secondary outcomes of the study include a comparison of mental health, as well as measures of app usability and acceptance.

Multiple self-report questionnaires are used to assess mental health. The German version of the Strengths and Difficulties Questionnaire (SDQ) (Rothenberger et al., 2008) is administered to capture self-estimated strengths and weaknesses on five different dimensions (emotional symptoms, conduct problems, hyperactivity/ inattention, peer relationship problems and prosocial behavior) with a total of 25 questions. Items are rated on a 3-point-Likert scale, ranging from ‘1 = not true’ to ‘3 = certainly true’. The SDQ shows acceptable psychometric properties, ranging from an internal consistency of Cronbach's *α* = 0.558 to 0.790 (Rothenberger et al., 2008). The overall well-being is assessed with the WHO-5 well-being index (WHO-5) (Brähler et al., 2007). The WHO-5 consists of five items which are rated on a 6-point-Likert scale ranging from ‘1 = all of the time’ to ‘6 = at no time’. The WHO-5 shows overall good psychometric properties with an internal consistency of Cronbach's *α* = 0.920 (Brähler et al., 2007). Ruminating thoughts are captured with the Brooding from Ruminative Response Scale (RRS Brooding) (Nolen-Hoeksema and Davis, 1999). In five items the RRS Brooding participants are asked to rate the extent of ruminative thoughts on a 4-point-Likert scale ranging from ‘1 = almost never' to ‘4 = almost always'. The RRS Brooding shows acceptable psychometric properties with an internal consistency of Cronbach's *α* = 0.780 (Schoofs et al., 2010). The applied Perceived Stress Scale 4 (PSS-4) (Klein et al., 2016) focuses on perceived stress based on four items that are rated on a 5-point-Likert scale. The PSS-4 has been proofed with overall good psychometric properties with *α* = 0.840 (Klein et al., 2016).

The German version of the Mobile App Rating Scale (MARS-G) (Messner et al., 2020) as well as self-generated items specifically tailored to the content of the MHG is administered to capture the app usability and acceptance of the MHG. The MARS-G is a self-report questionnaire consisting of a total of 29 items on four validated dimensions (engagement, functionality, aesthetics, subjective quality, and information quality), all of which are rated on a 5-point scale. The content of the specific selection options is adapted to the respective questions. The MARS-G shows acceptable to good psychometric properties, ranging from *ω* = 0.740 to 0.910 (Messner et al., 2020).

In addition, two outcomes (MDBF and DERS-SF) will be sent as short online questionnaires to all participants twice a day during the first week after completion of the baseline survey as part of an EMA. These two short instruments were chosen because they measure relatively unstable constructs, their repeated measurement should lead to a more reliable result and because they are time-efficient. The short form of the Multidimensional Mood State Questionnaire (MDBF) (Steyer et al., 1994) is included to investigate the participants' current well-being. The MDBF consists of 12 items on three bipolar dimensions (good-bad mood, awake-tired, calm-nervous), that are rated on a 5-point-Likert scale ranging from ‘1 = not at all’ to ‘5 = very’. Overall, the MDBF is characterized by good psychometric properties, ranging from an internal consistency of Cronbach's *α* = 0.730 to 0.880 (Steyer et al., 1994). For assessing emotion regulation, the short form of the Difficulties in Emotion Regulation Scale (DERS-SF) (Kaufman et al., 2016) is included. The DERS-SF covers 18 items in total, divided into six subscales (nonacceptance of emotional responses, difficulty engaging in goal-directed behavior, impulse control difficulties, lack of emotional awareness, limited access to emotion regulation strategies and lack of emotional clarity) that are rated on 5-point-Likert scale ranging from ‘1 = almost never' to ‘5 = almost always'. The DERS-SF will be conducted for both the individual questionnaire survey and the daily EMA survey. Overall, the DERS-SF indicated good psychometric properties, ranging from an internal consistency of Cronbach's *α* = 0.890 to 0.910 (Kaufman et al., 2016).

### 2.6 Statistical analysis

The main analyses will be performed in *R* (R Core Team, 2022). A mixed-measures analysis of variance (ANOVA) will be conducted with time (four levels: baseline, post, first follow-up, second follow-up) as the within-subjects factor and group (two levels: experimental, control) as the between-subjects factor. The model will contain independent variables for *Group* (coded 0 = CG and 1 = IG), *Time* (coded *Time* = 0, 1, 2, 3) and an interaction effect *Group* x *Time*. This analysis allows investigation not only of the main effects of time and group but also the interaction effect between time and group, which is crucial in understanding if the changes over time differed significantly between the two groups. In cases where significant interactions or main effects are found, post hoc analyses will be conducted to identify specific differences between time points and groups. Additionally, multi-level models with random intercepts and polynomial time variables will be estimated to evaluate differential longitudinal changes and potential non-linear pattern over time in the primary outcome (MHLS) across subjects in the treatment and control group (Hilbert et al., 2019).

In further exploratory analyses, the treatment effect will be investigated for the secondary outcomes in a similar fashion, and the moderating effect of app usage and acceptance on the treatment effects will be considered. Additionally, from the EMA data, properties of mood dynamics and emotion regulation competencies during everyday life will be derived using time-series analysis (e.g., autoregressive models and spectral analysis) and evaluated in their predictive capability for outcome measures (Schubert et al., 2021; Seizer et al., 2022).

Power analyses were performed in R 4.2 (R Core Team, 2022) using functionalities of the package *WebPower* (Zhang and Yuan, 2018). A previous meta-analysis that summarized results on the effectiveness of mental health literacy interventions in young people found moderate effect sizes (pooled *d* = 0.62) and average retention rates of about 86 % (Mills et al., 2023). Assuming the pooled effect size of previous interventions and a missing data rate of about 20 %, the study will allow performance of the planned analyses at a statistical power of over 80 % and an alpha error of 5 %. Full reproducible code and further information for the power analyses are available in the supplementary material and on OSF (https://osf.io/4wh5x/?view_only=040375cd3a56472597e788b97e5b8f28).

## 3 Discussion

This study aims to improve mental health in the young by providing a low-threshold, scalable, universal mHealth preventive intervention focusing on MHL and mental well-being. Hence, this project encounters the global peak for the onset of any mental disorder at an age of 14.5 years (Solmi et al., 2022) to prevent the development of mental disorders at an early stage (Weisz et al., 2005). MHealth interventions are particularly promising since they are potentially inexpensive, easily accessible (Paganini et al., 2018), and young people are especially familiar with how to use them (Robinson et al., 2016). To date, however, there are hardly any studies that have investigated the effectiveness and acceptance of mHealth-based preventive interventions. The aim of this study is therefore the scientific evaluation of an mHealth preventive intervention. We hypothesize, that the MHG will increase participants MHL. Further we hypothesize, that the MHG will have a positive impact on participants’ mental health. In addition, we expect a good usability and acceptance of the MHG. Assumptions will be tested in a randomised controlled study design with an intervention and a waiting list control group.

Based on previous studies on universal prevention of mental disorders (Hetrick et al., 2016; Loechner et al., 2018) we expect a rather small effect size. However, effects on knowledge of mental health may be higher (Schiller et al., 2014) and rather indirectly lead to a healthier behavior. Furthermore, in case of a negative live event that may exacerbate a mental illness, participants may be better informed about their emerging symptoms early on and search for professional support and treatment. Furthermore, general well-being may be improved due to the intervention by increased emotion regulation strategies and better stress coping skills. Thus, indirect preventive effects can be achieved. Despite the consistently lower impact of universal prevention programmes compared to selective prevention programmes, the benefits of targeting the whole population can be associated with reduced stigma and is a more comprehensive approach than focusing on a selected high-risk group. The use of digital technology, including EMA, to deliver mental health content is breaking new ground in prevention and process analysis. A more pessimistic view, however, would be that the provision of attractive information material by the MHG influences neither the knowledge nor the behavior of users. Participants who are already interested in such topics and already have prior knowledge may engage with the app's content. At the same time, it is possible that although knowledge is expanded, this does not lead to a change in behavior. In this case, the study would not show any effects.

The partially open recruitment has advantages and disadvantages. On the one hand, it enables a naturalistic sample consisting of more or less motivated users with more or less interest and previous psychological stress and knowledge. This means that realistic results can be expected. On the other hand, the power of the study depends on the room for improvement or change. If the majority of users already have a high MHL, the room for change will be small, which could lead to non-significant results. To at least partially address the problem, two recruitment methods were chosen: Recruitment in school classes and open recruitment on the Internet. A review of the descriptive data at the baseline time will show whether the samples differ. In addition, the range in which the MHL is already moving at the baseline measurement time will be examined.

Another limitation of the study is that the results are only reported on self-assessment. In addition, this is a self-help intervention where participants are asked to work through the app programmes on their own without the assistance of a therapist or coach. This comes with an increased risk of dropout but speaks to scalability and anonymity. As the young people are technologically savvy and used to acquiring digital content themselves, we evaluate this risk as moderate. Furthermore, this allows us to assess how engaging the content is as a stand-alone intervention. In this context, a further limitation is that it cannot be ruled out that participants use alternative sources to the MHG to improve their MHL. Another limitation is the short time of follow-up assessment due to financial constraints of the study. However, this is a general limitation in prevention research that requires the occurrence of negative events over time to evaluate how well participants would cope with a future stressor as a result of acquired resilience. In addition, usage of content will only be tracked for the sample but not for individual participants, since personalised data from the app usage will be kept separately from scientific evaluation data. The “time spent on the app” should be considered in future studies as a moderating variable. Despite these limitations, with this study, we make a substantial contribution to closing the research gap in e-health prevention research.

## 4 Conclusion

The results of this study will provide more information about the possibilities of using mHealth-based universal prevention to enhance the mental health literacy and mental well-being in a young population. This goal is very timely, as young people, in particular, were highly burdened due to the COVID19 pandemic. Universal prevention services that are cost-effective, widely available, and easy to reach are urgently needed.

## Trial status

Recruitment started in June 2023 and will be completed by the end of August 2023. Data Collection will be completed by April 2024. Data analysis has not yet begun.

## Funding

This study is financed by own funds (appointment funds Prof. Löchner).

## CRediT authorship contribution statement

JL, CG and IB conceptualized the study and its design, OK is mainly responsible for managing the study, recruitment and conducting the RCT with the support and supervision of IB and JL. OK wrote the first draft of this manuscript, that was supported by IB and JL. LS conceptualized the EMA assessment and supported OK with the simulation of data. CG reviewed the final version and commented to all parts of the manuscript.

## Declaration of competing interest

The authors declare that they have no known competing financial interests or personal relationships that could have appeared to influence the work reported in this paper.

## Appendix A Supplementary data

Supplementary material Image 1
