# Supplementary material for: Improving mental health by improving the mental health literacy? Study protocol for a randomised controlled evaluation of an e-mental health application as a preventive intervention for adolescents and young adults
Source: Internet Interv. 2024 Mar 7;36:100733. doi: 10.1016/j.invent.2024.100733 (PMC10950738; doi:10.1016/j.invent.2024.100733)
Supplement: Supplementary file 1 — Supplementary material [file mmc1.docx]

Supplementary Materials

*A MHG Modules in detailed description*

Module 1 #Body

The module is divided into the sections ‘healthy nutrition’, ‘exercise’, ‘sleep’, ‘breathing’ and ‘stimulants’. This module describes the connections and interactions between mental and physical health. The section ‘healthy nutrition’ explains the backgrounds of emotional eating and gives recommendations on how to implement a more mindful diet (e.g. eating more filling food that provides important nutrients instead of fast food). The sections ‘exercise’ and ‘breathing’ explain the positive impacts of movement and breathing for stress reduction and give easy practice tips (e.g. daily movement in nature, deep breathing for 20- 30 seconds). The section ‘sleep’ informs about sleeping hygiene and why the act of sleeping is essential for brains recovery. The section ‘stimulants’ informs about the mechanisms of action of alcohol, caffeine and nicotine and their interactions with cognition, behaviour and feelings.

Module 2 #Stress and resources

This module informs in its first section about neurocognitive aspects of stress and how stress reactions take place in the body. Further, stress management strategies (e.g. talking to colleagues, small exercises) are offered and early warning signs of too much stress are discussed. The second section educates about the definition of resources and asks the user specific questions to discover their personal resources.

Module 3 #Here and now

This module provides in the first section basic information on mindfulness as well as some mindful exercises to try. The second section educates how changes in life can encourage the development of crisis and mental illnesses and gives tips on how to handle changes adequately .

Module 4 #Circumstances

The module is divided into the sections ‘finances and consumer goods’, ‘geopgraphical and temporal circumstances’, ‘human’ and ‘mindset’. The section ‘finances and consumer goods’ educates about material stability and its interactions with mental health, as well as social norms to which they are subject. The section ‘geographical and temporal circumstances’ tries to create an awareness of the influence of e.g. seasons, big and small cities on mental health. It informs about the control you play in the perception of these circumstances and how to change them. The section ‘human’ takes a look to the human circumstances that form part of everybody’s life and asks some questions for more awareness, on who to keep in life. The section ‘mindset’ summarizes the previous chapters and aims to create an awareness that circumstances partly define us, but that the person can always partly control them.

Module 5 #Emotional intelligence

This module educates about basic emotions and their evolutionary use. Furthermore, the most important components for dealing with one's own emotions, perception, acceptance, and emotion regulation strategies (e.g. imagining emotions in colours, feeling the exact location of emotions in the body) are explained and how to help others dealing with their emotions.

Module 6 #Needs

This module gives fundamental information of human needs, among other things on Maslow’s hierarchy of needs and impulses to recognize and communicate own needs, e.g. by learning to say ‘no’.

Module 7 #Relationships with others

This module educates about roles in everyday life, e.g. as parents in the family, as workers in jobs, as well as the accompanying benefits, demands and possible impacts on the social environment. Various socially competent behaviours/ skills are presented and exercises for practicing them taught (e.g. reacting to criticism, starting a conversation). In addition, the handling of interpersonal conflicts is discussed, as well as the differences between types of forgiving are explained.

Module 8 #Relationships with me

This module educates about the differences between self-care, self-compassion, and self-worth, as well as underlying dogmas and beliefs are explained. In addition, recommendations for practicing more self-care and acceptance of one owns mistakes are given.

Module 9 #Crisis

This module explains different types of crises, e.g. trauma, developmental crisis (e.g. puberty, job loss, relocation), and psychosocial crisis, and presents various coping strategies (e.g. seeking understanding and support, creating and planning new daily structure). This module also includes an exercise for recognizing one’s own strengths and solution strategies for overcoming crises.

Module 10 #Psychoeducation

This module informs about the most common mental disorders and their symptoms: depression, anxiety disorder, eating disorder, alcohol abuse, psychoses, and personality disorders, and gives an overview of the treatment options available, e.g. psychotherapy and psychotropic medication and how to obtain them.

Module 11 #Communication

This module teaches about successful communication and miscommunication, as well as the rules of interpersonal communication (e.g. congruency of verbal and nonverbal cues, factual content vs. transported self-disclosure). Further the concept of ‘active listening’ is explained and the difference between hearing and active listening. Recommendations are given on how to communicate one’s own well-being as well as on how to ask someone about their mental health status.

Module 12 #Values, goals, purpose

The last module informs about life values, their benefits, and gives practical recommendations on how to find one owns values. Further, it teaches how to achieve goals on the basis of planning and implementing goals into everyday life, as well as their linking to the purpose of life and human being.
